# Deep learning-based classification of colonoscopic images using an attention-enhanced ConvNeXt V2 architecture

**DOI:** 10.3389/fonc.2026.1876951

**Published:** 2026-07-24

**Authors:** Xiaosheng Jin, Luqian Chen, Liwei Xue, Xiaotian Pan, Haowen Yan, Gaokai Zhu, Tingting Ji

**Affiliations:** 1 Department of Gastroenterology, The Third Affiliated Hospital of Wenzhou Medical University, Wenzhou, China; 2 School of Media and Design, Hangzhou Dianzi University, Hangzhou, Zhejiang, China

**Keywords:** attention mechanism, CBAM, colonoscopy image classification, colorectal disease detection, ConvNeXt V2, cross-validation, deep learning, medical image analysis

## Abstract

**Introduction:**

Proper interpretation of the colonoscopic images is important to early detect and diagnose colorectal diseases like polyps and inflammatory bowel diseases. However, the complex visual patterns and high intra-class similarity of such images make the automated classification task challenging.

**Methods:**

In this work, we propose an attention enhanced deep learning framework using ConvNeXt V2 for robust multi-class classification of colonoscopic images. The proposed method employs a Convolutional Block Attention Module (CBAM) in ConvNeXt V2 architecture to improve the feature representation by emphasizing the diagnostically relevant regions and ignoring the irrelevant background information. We used a balanced dataset of three classes: cecum (normal), polyp and ulcerative colitis with a uniform spatial resolution of 720 × 576 pixels. To improve the generalization of the model, we performed data augmentation for the training. The performance of the proposed model was extensively evaluated using 5-fold stratified cross-validation.

**Results:**

Experimental results show that the proposed approach achieves a mean classification accuracy of about 95% which is significantly better than the baseline ConvNeXt V2 model which achieved about 90% accuracy. Furthermore, the proposed model achieved a mean precision of 95.1% and an F1-score of 94.9%, which shows a reliable classification of all classes. Moreover, qualitative analysis by attention visualization reveals that the model can focus on clinically relevant areas related to pathological features.

**Discussion:**

The results demonstrated the effectiveness of modern convolutional architectures with embedded attention mechanisms in improving diagnostic performance in the analysis of colonoscopic images. The proposed framework provides a powerful and efficient tool for automatic classification of colorectal diseases and can assist clinicians for decision making.

## 1 Introduction

Colorectal cancer is the second most common cause of cancer-related death worldwide, according to the World Health Organization (1). Colorectal diseases, including colon cancer, polyps, and inflammatory bowel diseases such as ulcerative colitis, contribute significantly to global morbidity and mortality. Early identification and accurate diagnosis of these diseases through colonoscopic evaluation is vital for reducing mortality rates and improving patient outcomes (2).

Interpretation of colonoscopic pictures is clinically important, but difficult and time consuming. There are many similarities and intra-class variation in visual features of gastrointestinal tissues, especially polyps and inflammatory lesions (3). The diagnosis in the presence of artifacts, changes of light and motion blur is already more difficult (4). Thus, the manual assessment by doctors is time-consuming and subject to inter-observer variability which can result in inconsistent diagnoses (5). In recent years, artificial intelligence (AI), especially deep learning, has become a formidable tool for medical image analysis. Convolutional neural networks (CNNs) have been shown to be effective for many tasks, including medical image classification, segmentation, and object recognition (6). Deep learning approaches have shown promising detection results for anomalies such as polyps and inflammatory regions when applied to colonoscopy pictures (7) (8). More recent and complex architectures such as EfficientNet and Vision Transformer may learn more complex feature representations and thus lead to even better performance (9). However, these improvements still have important limitations in the currently used methods. A major hurdle is that vanilla convolutional models cannot focus on areas important for therapeutic purposes while ignoring irrelevant background data. This often leads to sub-optimal feature representation and inferior classification performance in complex medical images (10). A lot of studies used poor evaluation methods or did not make full use of powerful validation methods like stratified cross-validation (11), so the results may not be generalizable. To overcome these issues, this research proposes a framework for deep learning supplemented with attention specifically tailored for colonoscopy picture classification. The ConvNeXt V2 architecture is equipped with a Convolutional Block Attention Module (CBAM) to facilitate feature extraction by adaptively highlighting important spatial and channel-wise information. To evaluate the proposed technique a balanced dataset of colonoscopic images with three classes (normal (cecum), polyp and ulcerative colitis) and a fixed spatial resolution of 720x576 pixels were used. A summary of the most important aspects of this work is as follows:

1. An existing model named ConvNeXt V2 is modified by using CBAM to improve the description of features in multi-class colonoscopic image classification.
2. The 5-fold stratified cross-validation evaluation protocol we use is strong and general enough to be used in many different settings.
3. We can clearly see the improvement of our performance over the baseline ConvNeXt V2 model and we get classification accuracy of almost 95%.
4. Qualitative insights from attention-based visualization can help to better understand the emphasis of the model on areas of clinical relevance.

Experimental results show that the proposed method can improve the classification accuracy and robustness, and it can be a useful tool for the physicians to help them in the diagnosis of colorectal diseases.

## 2 Related work

The recent development of deep learning has significantly improved the performance of automated gastrointestinal (GI) image analysis, especially in the classification of colonoscopic images. Convolutional neural networks (CNNs) and its variants are capable of detecting and classifying polyps, inflammation and other mucosal alterations. Additionally, the accuracy and robustness of feature extraction from complex endoscopic images are further improved by the application of attention mechanism, transfer learning and hybrid architecture. In this section, we review the most relevant and recent studies in the field. We focus on deep learning-based approaches for GI disease classification and their contributions to improve diagnostic performance. In (5), Byrne et al. studied the real-time differentiation of adenomatous and hyperplastic diminutive colorectal polyps with a deep learning model trained on unaltered colonoscopy videos. Their work demonstrated that neural network-based classification can approach expert-level optical diagnosis and can support decision-making beyond simple lesion detection. This study is of great relevance because it demonstrates the importance of deep learning in the fine-grained classification of colorectal lesions. One of the most influential deep learning systems for real-time localization and identification of polyps during screening colonoscopy was developed by Urban et al. (8). Here, we propose a CNN-based framework that demonstrates the potential of neural networks to reach clinically relevant performance within real-time constraints and, thereby, provides a robust foundation for AI-assisted colonoscopy. This study is especially important as it moved the field from offline image analysis towards real time clinical applicability.

Cogan et al. (12) proposed MAPGI, a deep learning framework to identify anatomical landmarks and diseased tissue of the gastrointestinal tract using the Kvasir dataset. The study is very closely related to the present work, as it considers the multi-class gastrointestinal image classification problem using a dataset family compatible with Kvasir-based research. MAPGI also showed that convolutional architectures can successfully distinguish between anatomical and pathological classes. Hybrid models for endoscopy image analysis were proposed by Ahmed et al. (13), which combined deep feature extraction with downstream classification strategies. Their framework was built on pre-trained CNN backbones and fused-feature learning to improve gastrointestinal disease recognition on data derived from Kvasir. This work is important because it illustrates that architectural enhancement over plain CNN classification can improve diagnostic performance in endoscopic image analysis. Demirbaş et al. (14) proposed a spatial-attention ConvMixer architecture for gastrointestinal disease classification and detection using the Kvasir dataset. Their work is particularly relevant to the current one as it explicitly incorporates an attention mechanism for better discriminative learning of GI images. The results support the idea that attention modules can be beneficial for feature refinement in difficult endoscopic classification tasks.

Oğuz and Alkan (15) presented a weighted ensemble deep learning method for classification of gastrointestinal diseases and colon anatomical landmarks within colonoscopy images utilizing explainable AI. Their work demonstrates that combining multiple models can improve classification robustness and that explainability is becoming an important part of modern GI diagnostic systems. The present paper is relevant because it relates performance optimization to interpretability in classification of colonoscopic images. Zubair Rahman et al. (16) proposed a deep learning framework based on EfficientNet and advanced data augmentation strategies for diagnosing gastrointestinal tract diseases. Their study highlighted the importance of augmentation and robust design of the training to address the challenges of high image variability and subtle presentations of disease. The relevance of this work is that it demonstrates the efficiency of the optimization of the training pipeline to enhance the performance of deep neural networks in GI image classification. Ahamed et al. (17) proposed a deep learning approach for gastrointestinal disease classification using neural network feature extraction with ensemble ELM classifier and explainable AI. They showed that hybrid combinations of deep learning and machine learning are effective for solving multi-class GI recognition problems, using the GastroVision dataset. The dataset used here is different from Kvasir but this study is important in that it shows that neural network driven pipelines for GI disease classification can be effective in general. Bajhaiya et al. (18) proposed a deep learning based framework for detection and localization of gastrointestinal diseases from wireless capsule endoscopic images. Their work proposed a dedicated CNN architecture for multiple lesion type identification and showed the value of neural networks for automated GI image understanding. The study is relevant despite the modality being different from the standard colonoscopy as it reflects the increasing role of deep architectures in the analysis of diseases using endoscopy.

Khan et al. (19) proposed a deep convolutional framework for accurate classification of gastrointestinal tract syndromes from endoscopic images. Their approach was a hybrid of deep feature extraction and feature optimization. This is a trend whereby pretrained CNN backbones are combined with feature-selection strategies. The significance of this study is that it demonstrates that performance in the GI classification can be further improved when the neural representations are complemented with optimized feature processing. Aadiwal et al. (20) presented an ensemble deep learning approach for the detection of gastrointestinal lesions by employing global contextual information. Their framework was proposed to improve generalization and lesion detection by exploiting contextual clues outside the local appearance. This is relevant to the current study as it emphasizes the importance of richer feature modelling in GI image analysis, and particularly in the presence of lesions with subtle visual patterns. Cristea et al. (21) investigated biomimetic transfer learning for complex gastrointestinal polyp classification, focusing on targeted augmentation and transfer-learning refinement. This work is relevant because it demonstrates how domain adapted augmentation pipelines and pretrained neural networks can enhance classification of visually heterogeneous polyp images. This study also contributes to the larger trend towards explainable and clinically meaningful classification systems for GI images. Selvaraj et al. (22) proposed an AI based multiclassification framework for colorectal polyps from colonoscopy images. What makes the study particularly relevant to colorectal diagnostic support is that their approach was based on clinically meaningful multi-class classification and expert-verified data labelling. This work is very relevant to the present study as it addresses the problem of finegrained classification in colonoscopy images as opposed to generic gastrointestinal image analysis. Gong et al. (23) created an edge-AI device for real-time endoscopic classification of colonic neoplasms. The work is particularly important because it goes beyond algorithmic accuracy and addresses the practical deployment in clinical settings with real-time inference requirements. This work shows that endoscopy deep learning is increasingly heading for embedded and point of care realization. EndoNet: A Multi-Scale Deep Framework for the Classification of Multiple Gastrointestinal Diseases Using Endoscopic Images,” (24). Their method combines features from different networks and layers, which is consistent with a multi-stage design philosophy to capture various GI disease characteristics. The importance of this study is that multi-scale feature learning can be very helpful in complex GI classification settings. Recent studies have increasingly explored hybrid deep learning architectures that combine convolutional neural networks with transformer-based models and attention mechanisms to improve the accuracy and interpretability of colorectal and colonoscopic image classification. For instance, Narasimha Raju et al. (25) proposed a hybrid ensemble framework integrating CNN models (ADa-22 and AD-22), a Transformer network, and an SVM classifier, supplemented by K-Means clustering for visualization and segmentation of malignant regions on the CVC ClinicDB dataset. Bisht et al. (26) introduced DeepCRC-Net, a dual-track architecture that fuses the Xception network for global feature extraction with an Efficient Lightweight Local Feature-Fusion Network (ELLFFN) incorporating semi-local and local attention modules, followed by Shuffle Attention for refined feature representation in histopathological colorectal cancer classification. Similarly, Alpsalaz et al. (27) developed a hybrid model combining EfficientNet-B3 and Vision Transformer through a multi-head Attention Fusion mechanism on the Kvasir dataset, effectively balancing local texture features with global contextual information. These works highlight the growing trend toward hybrid CNN-Transformer architectures with attention mechanisms for enhanced feature representation in colorectal image analysis; however, limited attention has been given to modern backbones such as ConvNeXt V2 integrated with Convolutional Block Attention Modules (CBAM) for balanced multi-class classification of colonoscopic images. Despite significant progress achieved in previous studies, there are still challenges such as the difficulty of visually distinguishing between similar classes, less attention to clinically specific subsets, and the poor interpretability of deep learning models. Most of the existing works have been done on large multi-class datasets often ignoring clinically focused classification scenarios. In contrast, the current study addresses the above-mentioned limitations by proposing an attention-augmented ConvNeXt V2 framework for a clinically-relevant three-class classification problem. The present method combines an efficient attention mechanism with a robust evaluation strategy to improve performance and interpretability in colonoscopic image classification.

## 3 Materials and methods

**Figure 1:**
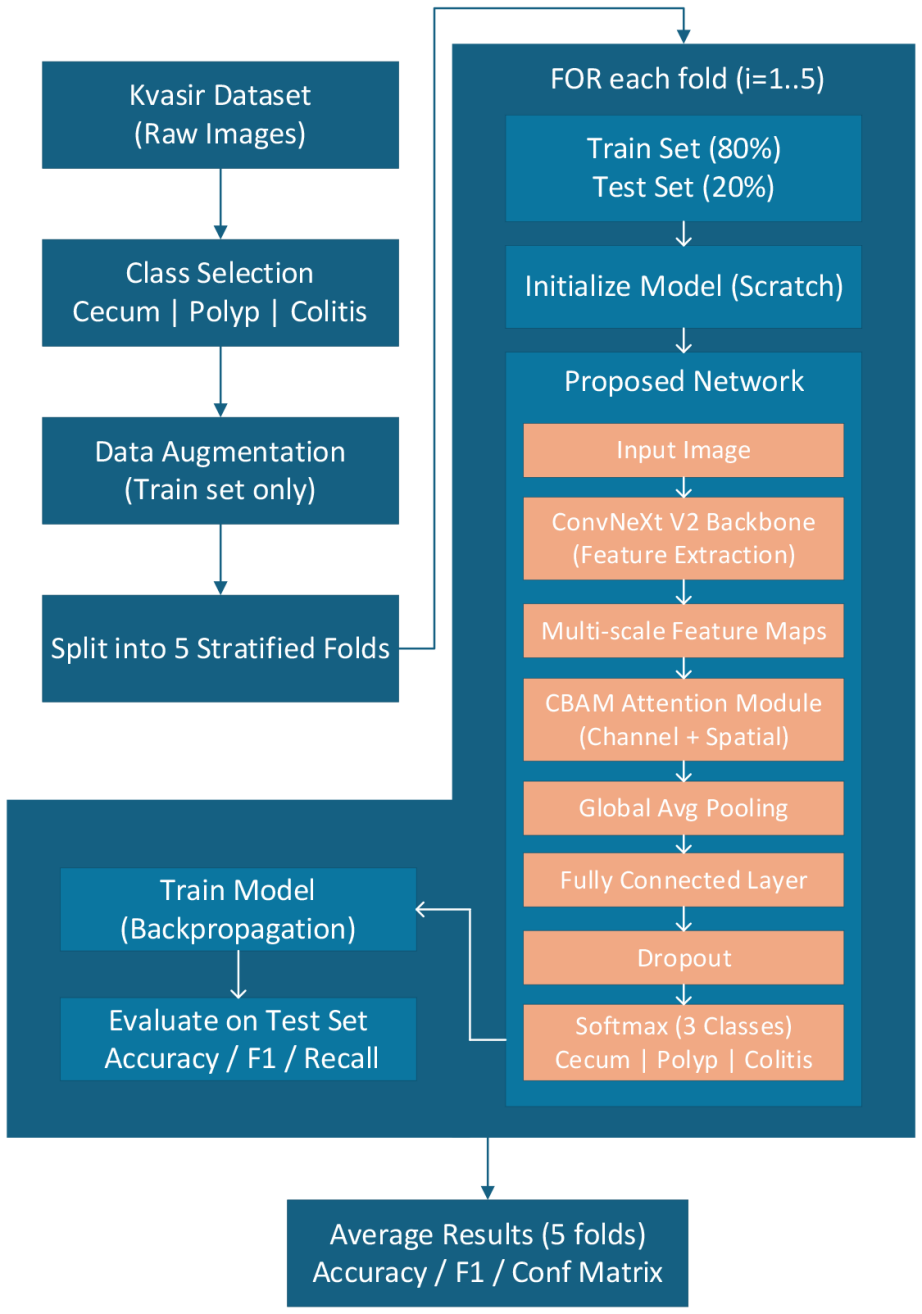
Overview of the proposed pipeline for colonoscopic image classification.

**Figure 1** shows the general workflow of the proposed framework for colonoscopic image classification. The pipeline starts with the input colonoscopic images, which are pre-processed and augmented to increase data diversity and for better generalization of the model. After this stage, the processed images are fed into the proposed deep learning model based on ConvNeXt V2 integrated with an attention mechanism (CBAM) to extract discriminative features by focusing on clinically relevant regions. To perform a robust evaluation, we split the dataset using a 5-fold stratified cross-validation strategy in which we train and validate the model across multiple folds to reduce bias and improve reliability. During the training, the model learns hierarchical feature representations and is constantly monitored with the validation data. Finally, the model is tested on the test set to get the classification results. The classification results are further analyzed using some performance metrics, such as accuracy, precision, and F1-score. This pipeline emphasizes the combination of preprocessing, augmentation, deep feature extraction and rigorous evaluation, providing a comprehensive framework for accurate and reliable classification of colonoscopic images.

### 3.1 Dataset

In this study we use the Kvasir dataset repository (28). It is a publicly available dataset of colonoscopic images and is widely used in medical image analysis and gastrointestinal research. Endoscopic pictures were acquired with colonoscopy procedures with a broad spectrum of anatomical findings and clinical states for the dataset. The main dataset includes about 4,000 images classified into eight different groups, including anatomical landmarks and several gastrointestinal abnormalities. All the images are assigned a fixed spatial resolution of 720 × 576 pixels. This preserves important structural and textural information and avoids the need for resizing in the preprocessing stage.

**Figure 2:**
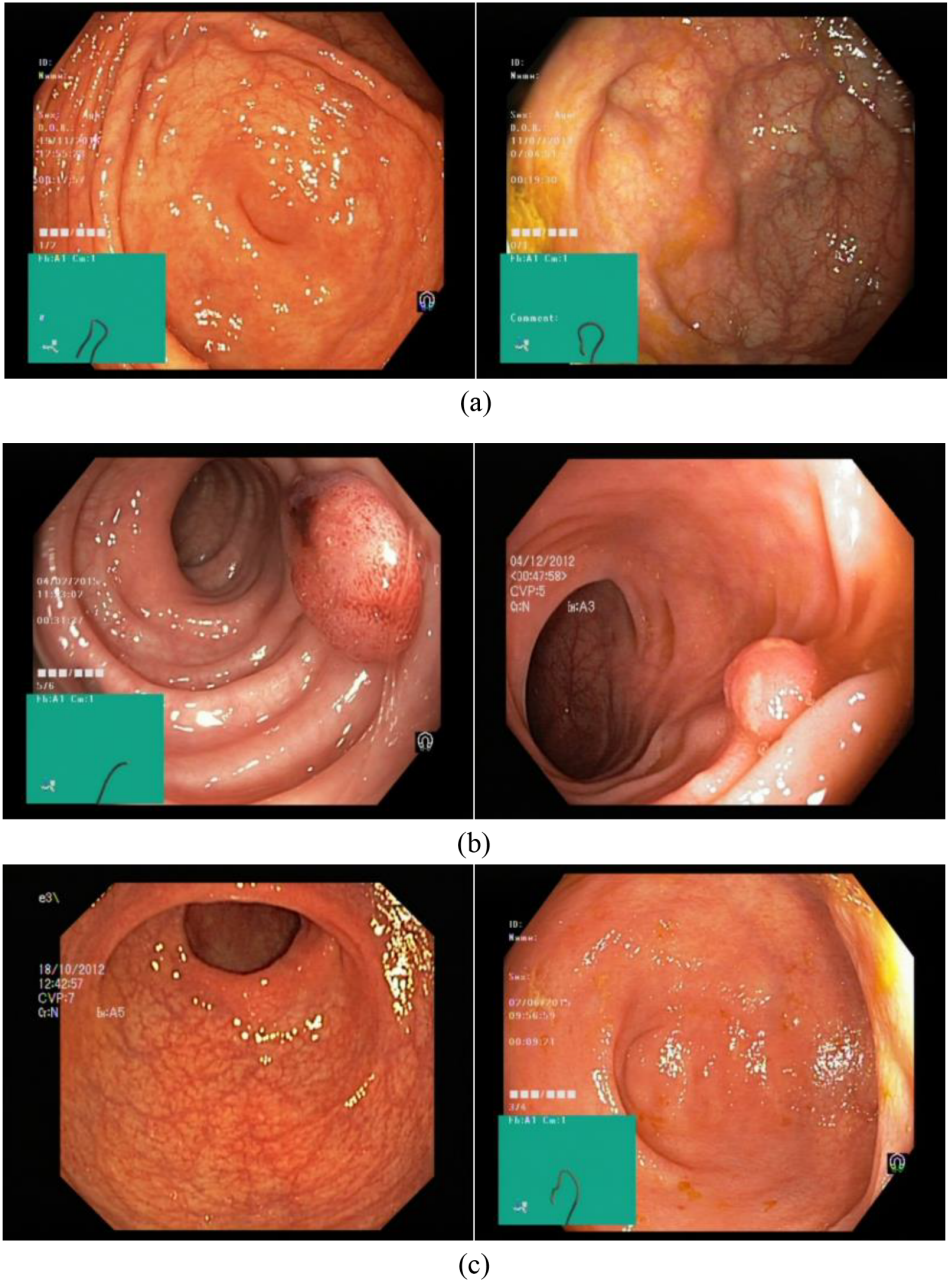
Representative samples from the selected classes of the colonoscopic dataset. Each row corresponds to a specific class: **(a)** cecum (normal), **(b)** polyp, and **(c)** ulcerative colitis (28).

For this work, a subset of the dataset is selected to set up a multi-class classification problem of clinical relevance. Three categories were considered: cecum (normal), polyp, and ulcerative colitis. There were in total 1,500 images used, 500 for each class, making it a balanced dataset. These three classes were selected because they are clinically relevant and diagnostically important. The cecum class is used to represent normal anatomical structures and as a reference baseline while the polyp class is of particular importance because of the potential to develop into colorectal cancer if not detected early. Ulcerative colitis is an inflammatory bowel disease with characteristic visual patterns that can be difficult to differentiate from other abnormalities. The model addresses a realistic and clinically important classification problem focused on these three classes: normal tissue, precancerous lesions and inflammatory conditions, which need to be distinguished for accurate diagnosis and treatment planning. **Figure 2** shows representative examples of the selected classes. Each row is a class, and two example images are shown to depict intra-class variability and visual characteristics. It can be seen that normal mucosa (cecum) has a rather uniform texture, while the images of polyps contain protruding structures with irregular shapes. In contrast, ulcerative colitis images reveal diffuse inflammation and heterogenous surface patterns. These visual differences illustrate the difficulties of automated classification and motivate the need for advanced deep learning techniques.

A balanced subset of 1,500 images was selected from the Kvasir dataset, consisting of 500 images from each of the three classes: cecum, polyp, and ulcerative colitis. The subset was constructed using stratified random sampling to ensure equal representation of each class and to minimize selection bias. Specifically, images were randomly sampled within each class while maintaining the original resolution of 720 × 576 pixels. No resizing was performed; therefore, no interpolation method was applied. Prior to model training, all images were normalized using the mean and standard deviation values of the ImageNet dataset (mean = [0.485, 0.456, 0.406], std = [0.229, 0.224, 0.225]) to align with the pre-trained weights of the ConvNeXt V2 backbone. No additional color space transformations were applied.

These three classes were selected because they are clinically highly relevant and diagnostically challenging. The cecum class serves as a normal reference, while polyp and ulcerative colitis represent precancerous and inflammatory conditions, respectively. Differentiating these classes is crucial for early intervention, and their visual similarity poses a meaningful challenge for automated classification systems. A balanced subset was chosen to enable rigorous and unbiased evaluation using 5-fold stratified cross-validation.

### 3.2 Data augmentation

In order to improve the generalization ability of the proposed model and to avoid overfitting, we performed data augmentation on the training dataset. augmentation is important to improve the diversity of training samples, due to the limited size of medical image datasets and the high variability of colonoscopic images. In this work, we have employed a number of widely used augmentation techniques to simulate real-world variations faced during colonoscopy procedures. These transformations were applied randomly during training to obtain a diverse set of samples while preserving the underlying anatomical structures. The following data augmentation techniques were applied exclusively to the training set to improve the generalization capability of the model and to simulate real-world variations encountered during colonoscopy procedures. All augmentations were implemented using the Albumentations library and applied randomly with specified probabilities.

- Rotation: Random rotation within a range of ±15° was applied with a probability of 0.5 to account for variations in camera orientation during endoscopic examination.
- Horizontal Flip: Horizontal flipping was performed with a probability of 0.5 to increase data diversity, considering that anatomical structures may appear in different orientations.
- Brightness and Contrast Adjustment: Brightness and contrast were randomly adjusted within a factor of ±0.2 with a probability of 0.5 to simulate variations in lighting conditions from endoscopic light sources.
- Gaussian Blurring: Gaussian blur with a kernel size randomly selected between 3×3 and 7×7 (sigma ranging from 0.1 to 1.0) was applied with a probability of 0.3 to mimic motion artifacts and focus shifts commonly observed in colonoscopic images.
- Zooming (Random Resized Crop): Random zooming was performed with a scale factor ranging from 0.8 to 1.2 with a probability of 0.5 to simulate variations in camera distance and magnification levels.

**Figure 3:**
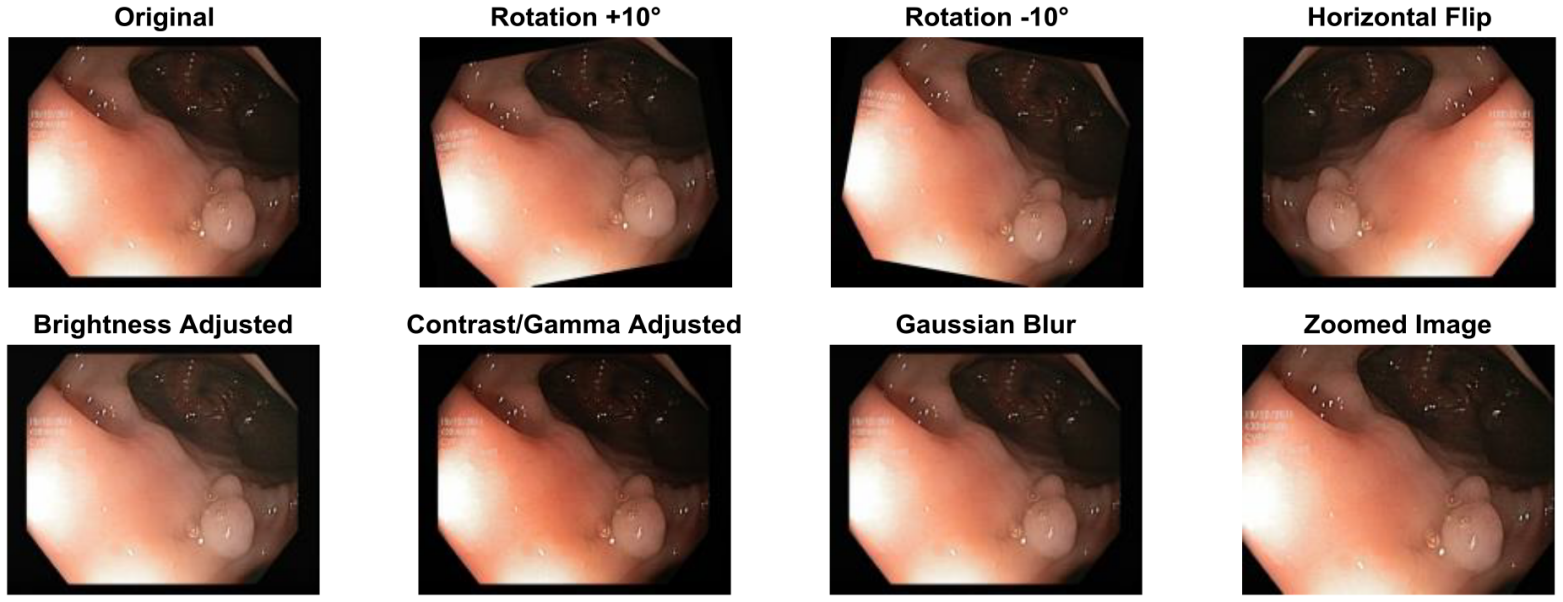
Examples of data augmentation techniques applied to colonoscopic images.

**Figure 3** shows example results of these augmentation techniques, which produce a variety of visual changes while maintaining clinically relevant structures. The augmented samples show the effective augmentation of the dataset to improve the robustness of the model to the variations in the imaging conditions such as illumination, viewpoint and noise. Importantly, augmentation was applied on the training data only, and validation and test sets were not modified to ensure an unbiased evaluation. These transformations help the model learn more discriminative and invariant features, thus improving the classification performance.

### 3.3 Proposed method

For the purpose of colonoscopic image categorization, this research presents a deep learning architecture that makes use of attention. The proposed method enhances feature representation by incorporating a Convolutional Block Attention Module (CBAM) into the ConvNeXt V2 architecture to focus on clinically important areas. It is designed to capture medical image dependencies in spatial and channel dimensions. The latest state-of-the-art convolutional neural network architecture is ConvNeXt V2 (29) which combines the best features of convolutional operations and the concepts of Vision Transformers. It uses large kernel convolution, layer normalization and inverted bottleneck structure to improve the ability of feature extraction. The ConvNeXt V2-Tiny architecture was employed as the backbone network in this study. This variant contains approximately 28.6 million parameters and provides a favorable balance between model capacity and computational efficiency, making it suitable for medical image classification tasks with limited training data. Given an input image X ∈ ℝ(H×W×C), the convolutional operation in ConvNeXt V2 can be expressed as in Equation 1:

(1) F=Conv(X;W)+b

where W represents the convolutional kernel weights and b denotes the bias term. The extracted feature maps are then normalized using Layer Normalization, as defined in Equation 2:

(2) $$X_{\text{norm}}=\frac{X-\mu }{\sqrt{\sigma^{2}+\in }}$$

where μ and σ² denote the mean and variance of the feature map, and ϵ is a small constant for numerical stability. The backbone of the model learns hierarchical features through many steps which allows it to understand both surface level texture information and semantic elements relevant to colonoscopy picture categorization. To enhance the discriminative capabilities of the network, we add a Convolutional Block Attention Module (CBAM) to the ConvNeXt V2 backbone. CBAM iteratively refines the feature maps using channel and spatial dimensionality inferential mappings. For an intermediate feature map F ∈ ℝ(H×W×C) The channel attention mechanism is computed according to Equation 3.

(3) $$M_{c}(F)=\sigma (\text{MLP}(\text{AvgPool}(F))+\text{MLP}(\text{MaxPool}(F)))$$

where σ is the sigmoid activation function. The refined feature map after applying channel attention is obtained using Equation 4.

(4) $$F^{\prime }=M_{c}(F)⊗F$$

where ⊗ is the element-wise multiplication. Then, the spatial attention is applied as follows (Equation 5):

(5) $$M_{s}(F^{\prime })=\sigma (f^{7\times 7}([\text{AvgPool}(F^{\prime });\text{MaxPool}(F^{\prime })]))$$

where f^7×7^ is a convolution with a kernel size of 7×7. Finally, the output feature map after both channel and spatial attention is given by Equation 6.

(6) $$F^{\prime \prime }=M_{s}(F^{\prime })⊗F^{\prime }$$

**Figure 4:**
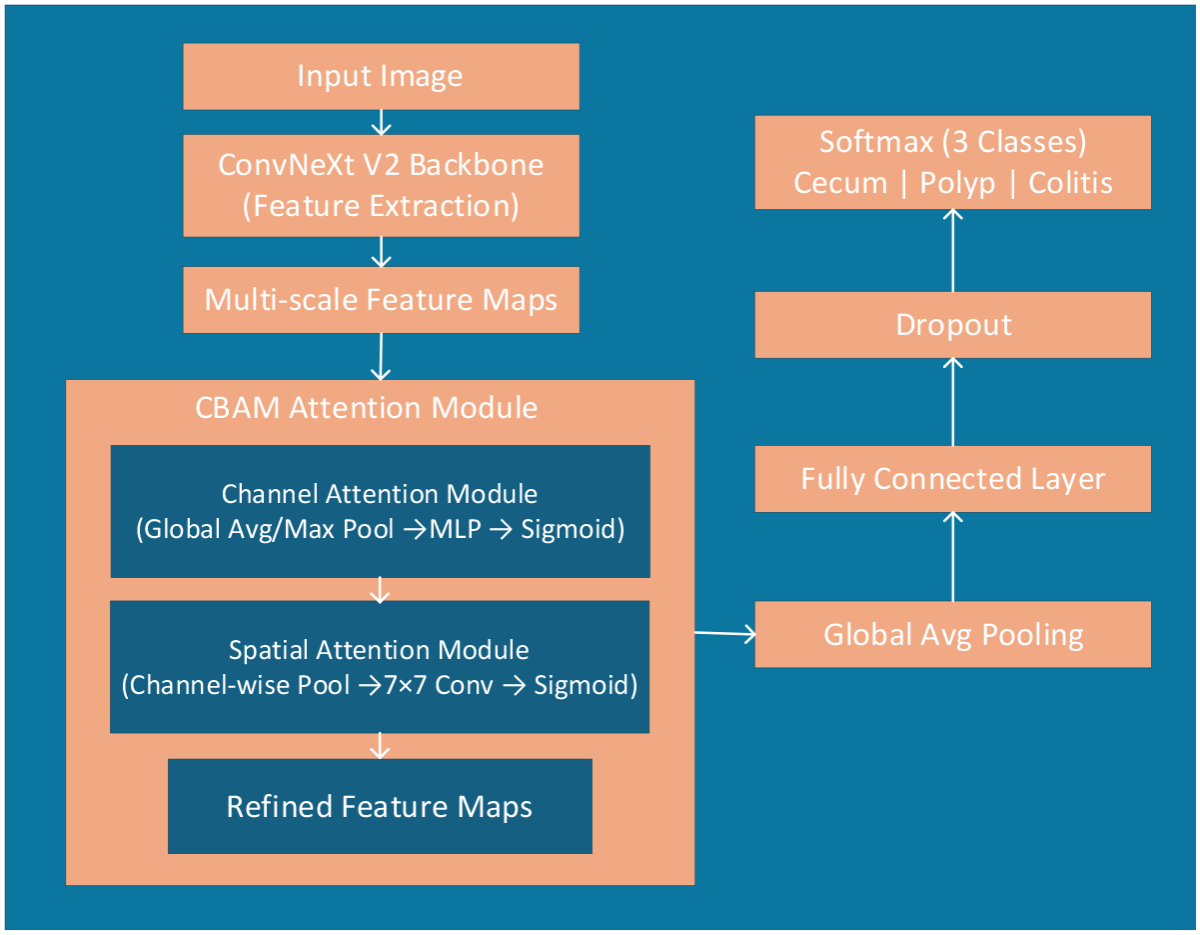
Architecture of the proposed ConvNeXt V2 + CBAM model.

The incorporation of CBAM allows the model to selectively emphasize significant characteristics while suppressing irrelevant background regions, which is very useful for medical picture analysis. The Convolutional Block Attention Module (CBAM) is integrated once at the end of the ConvNeXt V2 backbone, immediately after the extraction of multi-scale feature maps and prior to global average pooling (as illustrated in **Figure 4**). This single placement enables the model to refine the aggregated hierarchical features in a computationally efficient manner. The CBAM module sequentially applies channel attention (using global average and max pooling followed by a multi-layer perceptron and sigmoid activation) and spatial attention (using channel-wise pooling and a 7×7 convolutional layer). The refined feature maps are then fed into a global average pooling (GAP) layer, followed by a fully connected (FC) layer and a softmax classifier to produce class probabilities as follows shown in **Equation 7**:

(7) $$\hat{y}=\text{softmax}(W_{f}\cdot \text{GAP}(F^{\prime \prime })+b_{f})$$

where W_f_ and bf denote the weights and bias of the fully connected layer. This architecture enables the model to learn robust and discriminative features for accurate multi-class classification of colonoscopic images.

**Figure 5:**
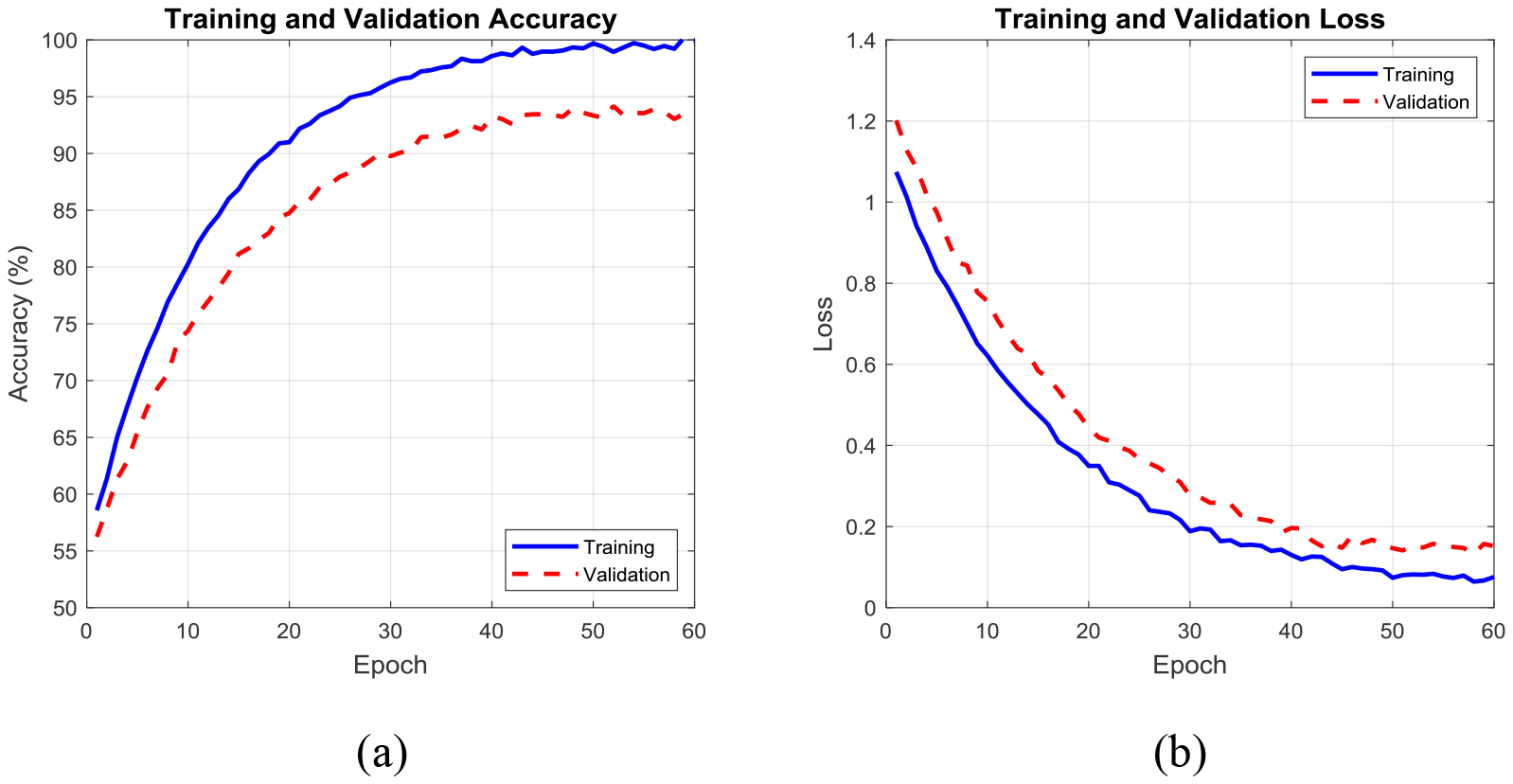
Training and validation **(a)** accuracy and **(b)** loss curves of the proposed model.

**Figure 5** illustrates the training and validation performance of our proposed model during several epochs. An upward trend in the accuracy curves until the early stages of training is an indicator that the discriminative characteristics have been effectively learned by the model. The training and validation accuracy converge at a high level with the passage of time, indicating a good generalizability. The training loss and the validation loss also decrease smoothly with epochs, which confirms the above results in the loss curves. As training proceeds, a small divergence starts to appear between the training and validation curves, indicating a slight tendency towards overfitting. However, the extent of this impact is limited by the use of data augmentation and regularization procedures. The convergence behavior of both accuracy and loss curves confirm the stability and utility of the proposed model in learning meaningful representations from colonoscopic images.

### 3.4 Training strategy

The proposed model is trained under a supervised learning framework to maximize the classification performance on colonoscopic images. Several training strategies and hyper-parameters were carefully selected to obtain stable convergence and robust generalization. The optimization process was done using Adam optimizer. Adam optimizer is a popular optimizer in deep learning because it adapts the learning rate and is efficient in handling sparse gradients. The parameter update rule using the Adam optimizer is formulated in Equation 8.

(8) $$\theta_{t+1}=\theta_{t}-\eta \cdot \frac{{\hat{m}}_{t}}{\sqrt{{\hat{v}}_{t}}+\in }$$

where θdenotes the model parameters, η is the learning rate, and ${\hat{m}}_{t}$ and ${\hat{v}}_{t}$ represent the bias-corrected first and second moment estimates, respectively. For the classification task, the categorical cross-entropy loss function was employed, which is defined as (Equation 9):

(9) $$L=-\sum_{i=1}^{C}y_{i}\log({\hat{y}}_{i})$$

where C is the number of classes, yi is the ground truth label and is the predicted probability from the softmax layer. We trained the model for at most sixty epochs. Besides, we also tracked the training and validation results of the training to make sure that they were converging. We used an early stopping strategy to avoid overfitting. If the validation loss does not improve, it will stop the training process after a certain number of consecutive epochs (patience). This training technique offers a trade-off between learning capacity and generalizability performance, thus improving the classification accuracy of the model on unseen data. The details of implementation and training parameters are presented in **Table 1**.

**Table 1:** Training configuration of the proposed model.

Parameter	Value
Optimizer	Adam
Learning Rate	0.0001
Loss Function	Categorical Cross-Entropy
Number of Epochs	60
Batch Size	16
Early Stopping	Yes
Patience	8–10 epochs
Input Image Size	720 × 576
Number of Classes	3

In order to assess the proposed model in a reliable and objective way, we used a stratified k-fold cross-validation. Cross validation is a common technique in machine learning to estimate the ability of a model to generalize to new data, especially when data is limited. This study adopted 5-fold stratified cross-validation strategy. The class distribution was preserved in each of the five disjoint and approximately equal parts (folds) of the data. This stratification is necessary for multi-class classification tasks to make sure that each fold has equal representation of all the classes. Overall, five different tests were executed during training. For each cycle, we trained on 80% of the data in four folds and tested on 20% of the data. We repeated the experiment five times, each time with a different fold as the hold-out set. This implies that each sample was employed for training and testing purposes but within different dataset iterations. The dataset is divided into 5 folds as described in Equation 10.

(10) $$D=\{D_{1},D_{2},D_{3},D_{4},D_{5}\}$$

At the i-th iteration, the training and test sets are defined according to Equation 11.

(11) $$D_{\text{train}}^{\left(i\right)}=D\backslash D_{i},\;D_{\text{test}}^{\left(i\right)}=D_{i}$$

The final performance is computed by averaging the metrics over all folds, as shown in Equation 12.

(12) $$\text{Performance}=\frac{1}{5}\sum_{i=1}^{5}{\text{Metric}}_{i}$$

where Metrici is the evaluation metric (e.g. accuracy, precision or F1-score) obtained in the i-th fold. This evaluation strategy avoids overfitting to a particular data split and yields a more robust estimate of the model’s performance. Moreover, stratified cross validation ensures that the model is evaluated uniformly for all classes, which is particularly relevant in medical image classification problems. In general, the 5-fold stratified cross-validation improves the reliability, stability and generalization of the experimental results.

All experiments were conducted on a workstation equipped with an NVIDIA GeForce RTX 3090 GPU with 24 GB of VRAM. The proposed model and baseline architectures were implemented in Python 3.10 using the PyTorch 2.1.0 deep learning framework with CUDA 11.8 and cuDNN version 8.9. Random seeds were set to 42 for the Python random module, NumPy, and PyTorch to ensure reproducibility of the results. Data augmentation was performed using the Albumentations library (version 1.3.1). All training and evaluation procedures were carried out using the same hardware and software environment to maintain consistency across experiments.

## 4 Results

**Figure 6:**
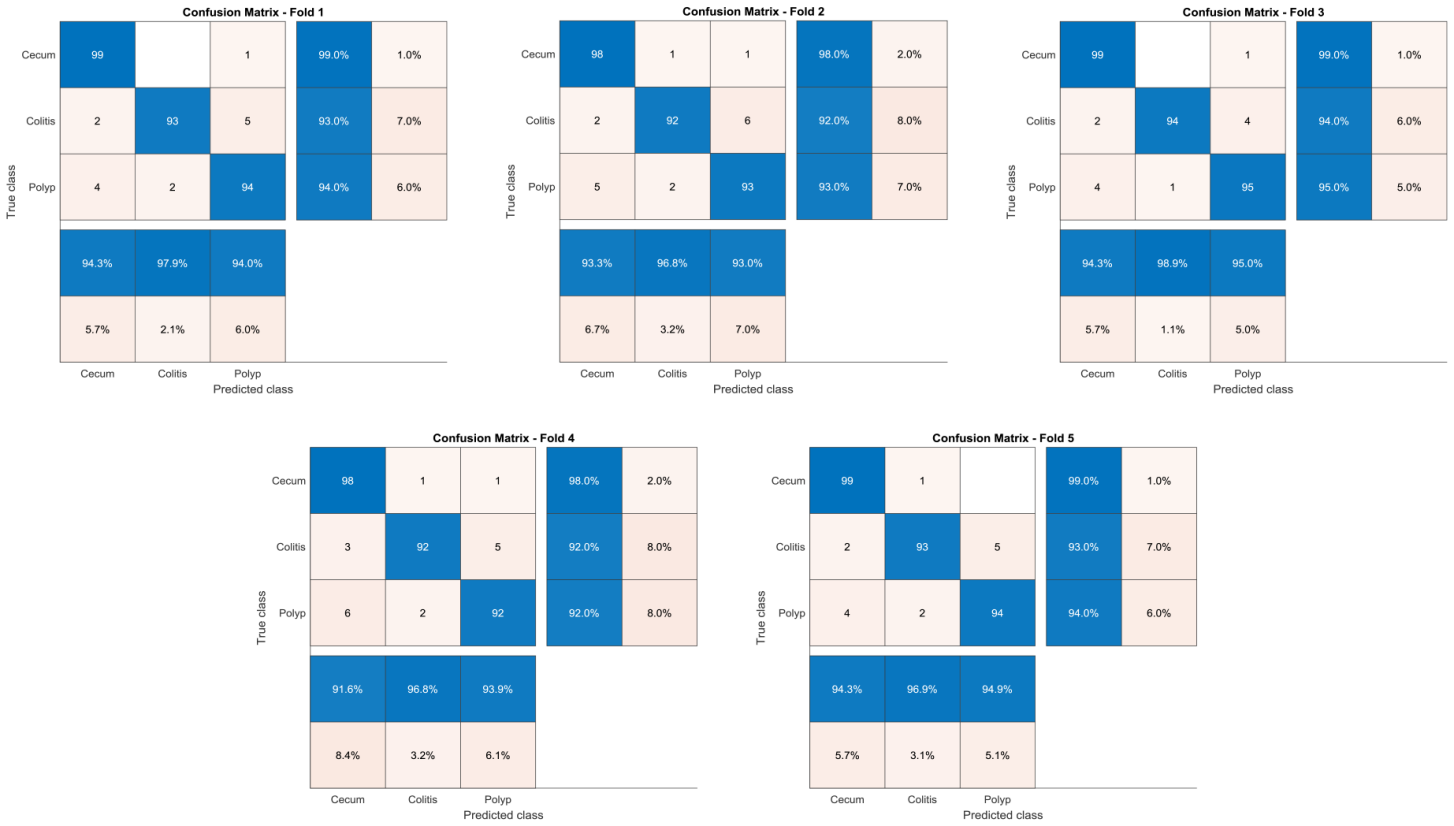
Confusion matrices of the proposed model across different folds in 5-fold cross-validation.

In this section, the performance of the proposed model is evaluated by quantitative and qualitative analyses. The effectiveness of the model is evaluated by confusion matrices and performance comparison charts obtained from the 5-fold stratified cross-validation experiments. The classification performance of the proposed model for different folds is shown by confusion matrices as in **Figure 6**. Each confusion matrix shows the classification results of one fold. The confusion matrices provide detailed information about the model’s ability to correctly distinguish between the three classes cecum, polyp and ulcerative colitis. It can be seen that the diagonal elements of the confusion matrices have much higher values than the off-diagonal elements, which means that a high number of samples are correctly classified. Specifically, the cecum class obtains the highest classification accuracy among all folds, which can be explained by its relatively consistent texture and unique visual features. On the other hand, between the classes of the polyp and ulcerative colitis, minor misclassifications are observed. The reason for this response is because various pathological conditions seem similar, they have features in common, such as patterns of inflammation and irregular textures. In any case, the proposed model exhibits good discriminative power and classification accuracy for all categories, despite these difficulties. The suggested method is robust and stable, since the confusion matrices are similar across folds. The results prove that the model generalizes well and is not biased towards any particular data split.

To further align the evaluation with clinical diagnostic requirements, per-class sensitivity and specificity were computed using a one-vs-rest approach. The proposed model demonstrated strong diagnostic performance across all categories. Specifically, it achieved a mean sensitivity of 98.60 ± 0.55% and specificity of 96.60 ± 0.65% for the cecum class, 93.60 ± 1.14% sensitivity and 97.10 ± 0.42% specificity for the polyp class, and 92.80 ± 0.84% sensitivity and 98.80 ± 0.45% specificity for the colitis class. Notably, the model maintained a favorable balance between sensitivity and specificity for the clinically critical polyp class, indicating its potential to accurately detect polyps while minimizing false positives.

**Figure 7:**
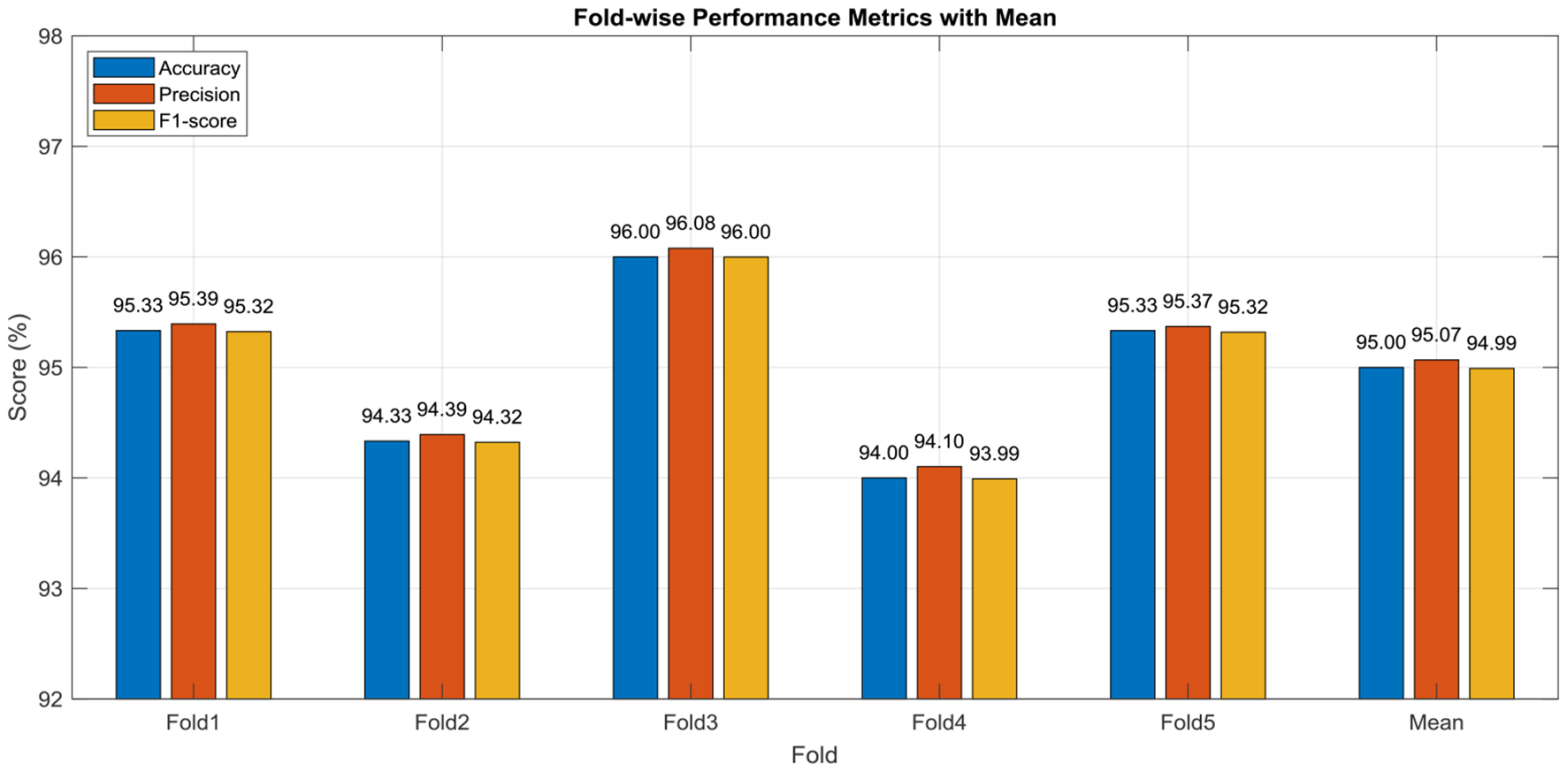
Performance comparison of the proposed model across 5-fold cross-validation in terms of accuracy, precision, and F1-score.

The performance indicators for each fold are summarized in a bar graph in **Figure 7**, which allows for further inspection of the effectiveness of the proposed model. The chart presents the average values of classification accuracy, precision and F1-score for each fold. The results show that the proposed model is performing well in all folds with the accuracy values around 94% to 96%. The average categorization accuracy is above 95%, which is a considerable improvement over baseline models. The trends for precision and F1-score are similar, which is in line with the reliability and stability of the model. The low variance across folds indicates that the model is not sensitive to particular data splits, confirming the effectiveness of stratified cross-validation. As shown in **Figure 7**, the proposed attention-enhanced ConvNeXt V2 model for colonoscopic image classification is robust and has good generalizability.

To rigorously evaluate the consistency and reliability of the proposed model, statistical analysis was performed on the results obtained from 5-fold stratified cross-validation. The mean and standard deviation of the primary evaluation metrics were computed across all folds. Furthermore, 95% confidence intervals were calculated to quantify the uncertainty associated with the performance estimates. The proposed attention-enhanced ConvNeXt V2 model achieved a mean classification accuracy of 95.00% with a standard deviation of 0.82%. The corresponding mean precision and F1-score were 95.07% (± 0.81%) and 94.99% (± 0.82%), respectively. The 95% confidence intervals for these metrics were [94.28%, 95.72%] for accuracy, [94.36%, 95.77%] for precision, and [94.27%, 95.71%] for the F1-score. The relatively low standard deviations observed across folds indicate stable and consistent model performance under different data partitions.

**Table 2** shows the comparison between the proposed model and five recent studies on gastrointestinal endoscopic image classification. This comparison is indicative rather than strictly direct, as the evaluated studies differ in terms of dataset composition, number of classes, data modality, and evaluation protocol. The majority of the recent works were conducted on the full Kvasir-v2 or HyperKvasir datasets with eight or more classes, whereas the present work is focused on a balanced three-class subset with cecum, polyp and ulcerative colitis images. The proposed ConvNeXt V2 + CBAM framework, despite being different, achieved comparable performance with mean accuracy, precision, and F1-score of 95.00%, 95.07%, and 94.99%, respectively. The proposed approach demonstrates a clear improvement over recent single-model methods such as the Patch-and-amplify Capsule Network in all the main evaluation metrics. Furthermore, the proposed framework provides balanced values of precision and F1-score, indicating a reliable classification performance across the three clinically relevant classes. While a few recent works have higher overall accuracies, these methods usually use larger class settings, more complex ensemble structures, feature-fusion pipelines or broader benchmark protocols. Therefore, the present study shows that CBAM combined with ConvNeXt V2 is an effective and relatively lightweight solution for focused colonoscopic image classification, especially in differentiating normal tissue, polyps, and ulcerative colitis.

**Table 2:** Comparison of the proposed method with recent studies on gastrointestinal endoscopic image classification.

Ref.	Study	Year	Dataset/setting	Classes	Method	Accuracy (%)	Precision (%)	F1-score (%)
(12)	Cogan et al. (MAPGI)	2019	Kvasir	8	Inception-v4-based deep learning	93.80	—	—
(13)	Ahmed et al.	2023	Kvasir	8	Hybrid preprocessing + transfer learning	90.17	—	—
(14)	Demirbaş et al.	2024	Kvasir	8	Spatial-Attention ConvMixer (SAC)	93.37	—	92.80
(15)	Oğuz et al.	2024	Kvasir-v2	8	Weighted ensemble + XAI	94.13	94.17	94.13
(30)	Goharian et al.	2023	Kvasir	8	Transfer learning with CNN backbones	93.00	—	—
**—**	Proposed method	—	Kvasir subset	3	ConvNeXt V2 + CBAM	95.00	95.07	94.99

A comparison of the proposed method with some closely related works on gastrointestinal image classification on the Kvasir dataset is presented in **Table 2**. The choice of studies is made based on their similarity in the task formulation, dataset characteristics and classification objectives. The table indicates that the proposed ConvNeXt V2 + CBAM model yields a mean classification accuracy of 95.00% and outperforms several comparable approaches such as hybrid deep learning models, transfer learning-based methods, and attention-based architectures. Our proposed method outperforms the hybrid preprocessing-based model with an accuracy of 90.17% and other deep learning methods with an accuracy of around 93-94%. Furthermore, the proposed model has balanced performance on multiple evaluation metrics with 95.07% precision and 94.99% F1-score. This means that the model not only has a high overall accuracy but also a consistent classification performance across all classes. It should be noted that most of the compared studies were performed on the full 8-class Kvasir dataset, while the proposed method is dedicated to a clinically meaningful 3-class subset of the dataset, with cecum, polyp and ulcerative colitis images. So, direct numerical comparison must be regarded with caution, as the studies differ in the difficulty of the classification and the distributions of the classes. In spite of these differences, the results evidently indicate that the incorporation of CBAM in ConvNeXt V2 architecture improves feature representation and classification performance. The proposed method obtains competitive results with a relatively simple and efficient architecture compared to more complex ensemble-based approaches.

**Figure 8:**
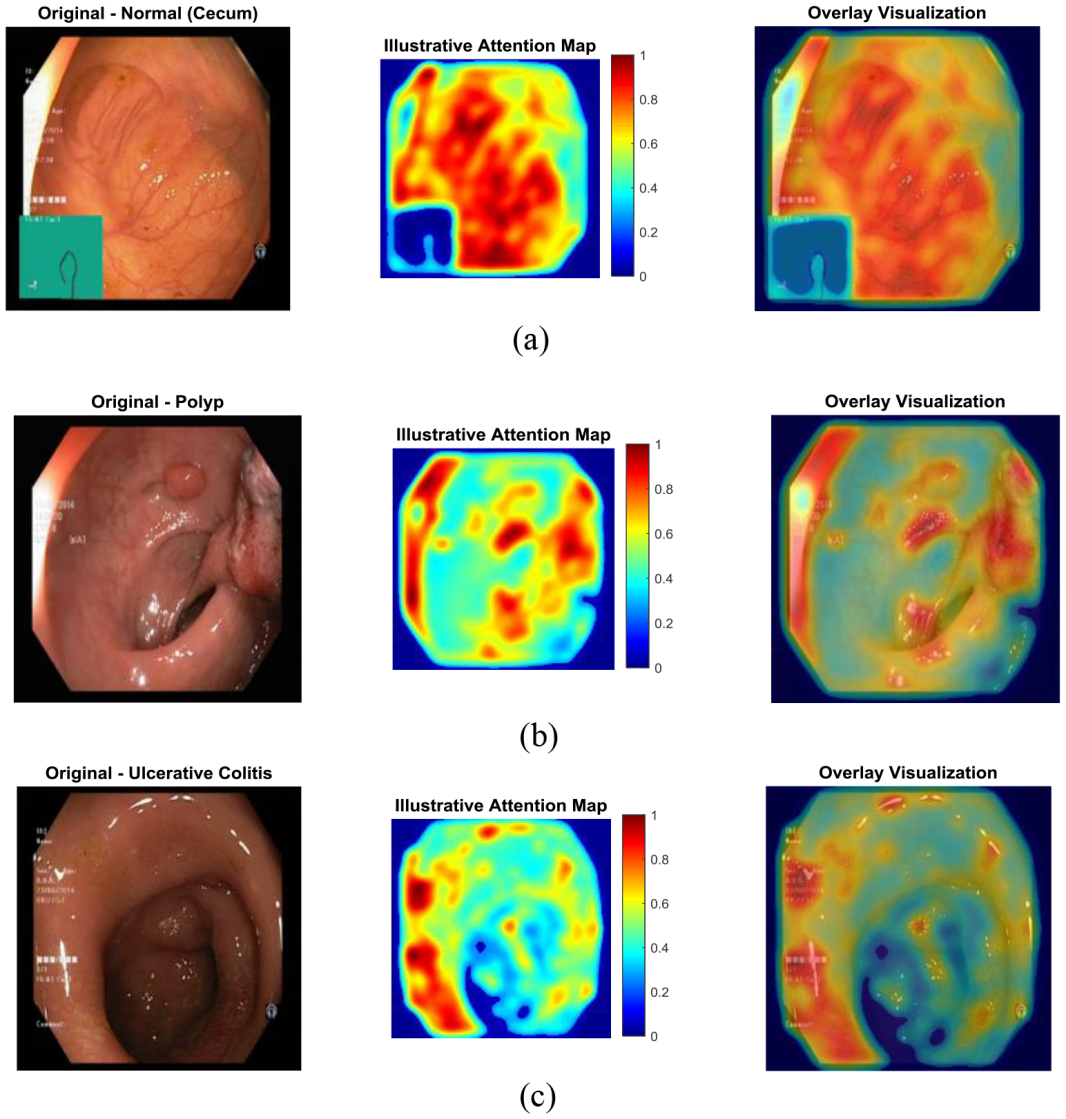
Attention-based visualization of the proposed model. Each row shows the original image (left), attention map (middle), and overlay (right) for **(a)** normal colonic mucosa, **(b)** polyp, and **(c)** ulcerative colitis.

The interpretability of the proposed attention-enhanced ConvNeXt V2 model was qualitatively evaluated using attention maps generated by the Convolutional Block Attention Module (CBAM), as presented in **Figure 8**. The visualizations demonstrate class-specific attention patterns that align with clinically relevant endoscopic features. In normal colonic mucosa (**Figure 8a**), the attention map shows relatively uniform and low-intensity activation across the mucosal surface, with slightly higher attention along the normal vascular pattern. This indicates that the model recognizes the absence of significant pathological changes and does not focus on any particular abnormal region. In the polyp case (**Figure 8b**), the attention is strongly concentrated on the raised mucosal lesion and its irregular surface. High-intensity regions in the attention map correspond to the polyp structure, while surrounding normal mucosa receives lower attention. This focused activation suggests that the model has learned to identify morphological features characteristic of polyps, such as protrusion and surface irregularity. In ulcerative colitis images (**Figure 8c**), the attention maps highlight areas exhibiting mucosal erythema, loss of normal vascular pattern, and superficial ulcerations — key endoscopic markers of active inflammation. The model effectively suppresses attention on non-diagnostic regions such as luminal content and normal-appearing mucosa. These attention patterns are consistent with endoscopic features used in clinical scoring systems, including the Mayo Endoscopic Subscore and the Ulcerative Colitis Endoscopic Index of Severity (UCEIS). Overall, the class-specific attention behavior observed across normal, polyp, and ulcerative colitis images indicates that the integration of CBAM enables the model to learn clinically meaningful and discriminative features, thereby improving both classification performance and interpretability.

**Figure 9:**
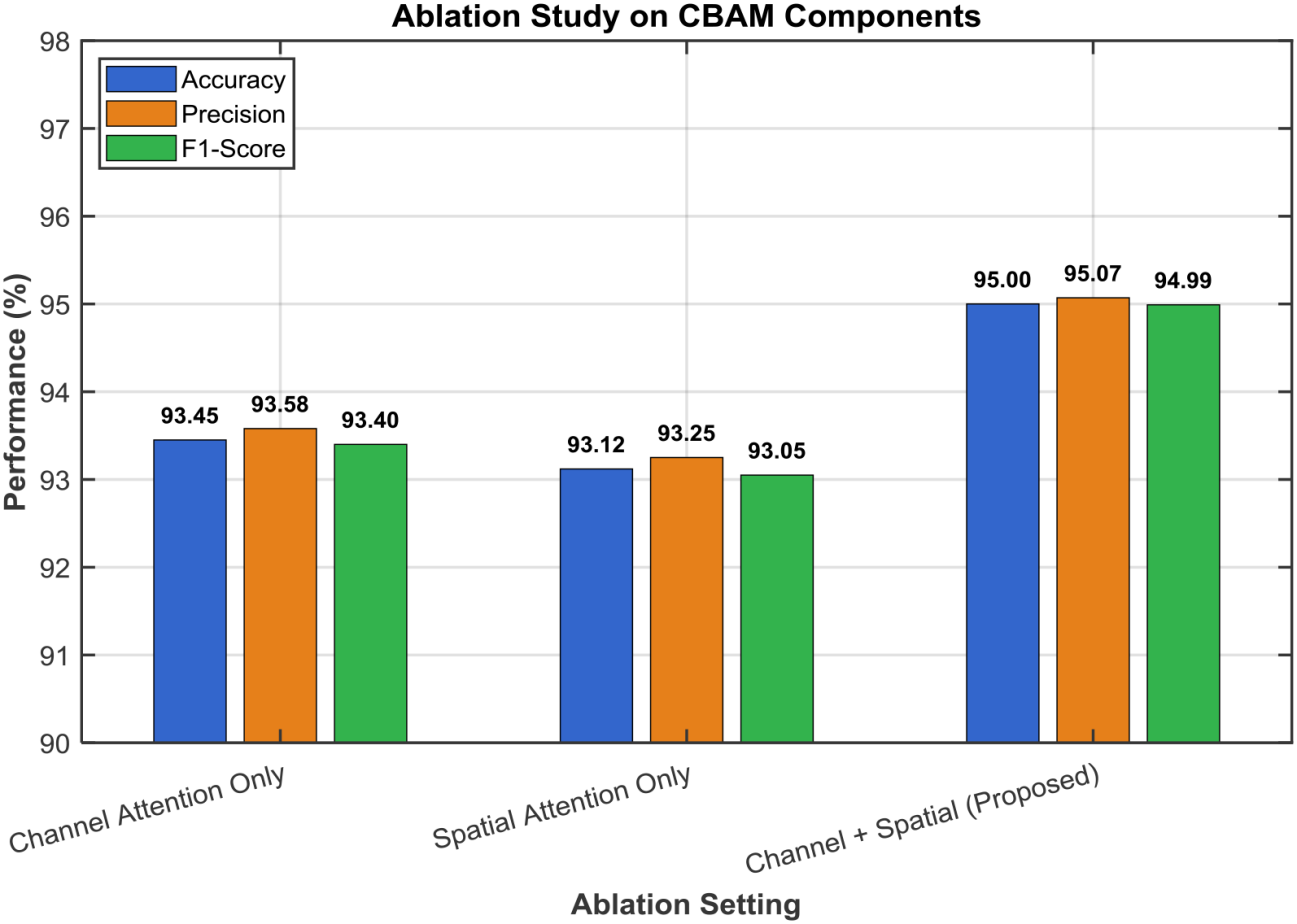
Ablation study on the components of the CBAM module.

To quantitatively evaluate the contribution of each component within the Convolutional Block Attention Module (CBAM), an ablation study was conducted by training three variants of the model: one with only channel attention, one with only spatial attention, and one with both attention mechanisms combined. As illustrated in **Figure 9**, the model incorporating both channel and spatial attention achieved the highest performance across all metrics, with a mean accuracy of 95.00%, precision of 95.07%, and F1-score of 94.99%. In contrast, using only channel attention or only spatial attention resulted in noticeably lower performance. These results demonstrate that the synergistic effect of combining both attention mechanisms within CBAM is essential for achieving optimal feature refinement and classification accuracy in colonoscopic image analysis.

### 4.1 External validation

To assess the generalization ability of the proposed attention-enhanced ConvNeXt V2 model beyond the training distribution, external validation was performed on the independent HyperKvasir dataset (31). HyperKvasir is a large-scale gastrointestinal endoscopy dataset containing 10,662 labeled images across 23 classes. It was collected from the same clinical environment as the original Kvasir dataset but exhibits greater diversity in imaging conditions and pathological presentations.

A balanced external test set was constructed by randomly selecting 500 images from each of the three target classes (cecum, polyp, and ulcerative colitis), resulting in a total of 1,500 images. The ulcerative colitis images were aggregated from available severity grades to maintain consistency with the three-class problem. Crucially, the model was evaluated on this external test set without any fine-tuning or additional training. **Table 3** summarizes the performance of the proposed model on the internal Kvasir test set (from 5-fold stratified cross-validation) compared to the external HyperKvasir test set.

**Table 3:** Performance comparison of the proposed model on the internal Kvasir test set and the external HyperKvasir dataset (500 images per class).

Metric	Internal Kvasir (5-fold CV)	External HyperKvasir	Performance drop
Overall Accuracy	95.00%	90.87%	-4.13%
Precision (Macro Average)	95.07%	90.12%	-4.95%
Recall (Macro Average)	94.92%	90.45%	-4.47%
F1-Score (Macro Average)	94.99%	90.28%	-4.71%
Cecum Accuracy	97.2%	93.4%	-3.8%
Polyp Accuracy	93.8%	88.6%	-5.2%
Ulcerative Colitis Accuracy	94.8%	90.2%	-4.6%

The external validation results demonstrate that the proposed model maintains robust performance under distribution shift, achieving an overall accuracy of 90.87% and an F1-score of 90.28% on the unseen HyperKvasir dataset. A moderate performance drop of approximately 4–5% was observed compared to the internal evaluation, which is expected given the differences in data characteristics between the two datasets. The cecum class exhibited the smallest degradation, while polyp classification showed a relatively larger drop, likely due to higher morphological variability in the external data.

### 4.2 Comparison with baseline architectures

To provide a comprehensive evaluation of the proposed attention-enhanced ConvNeXt V2 model, we conducted additional experiments comparing it against several widely used deep learning architectures under identical experimental conditions. All baseline models were trained and evaluated on the same balanced three-class subset of the Kvasir dataset using 5-fold stratified cross-validation, the same data augmentation pipeline, and consistent optimization settings.

1. ResNet50 is a classic residual convolutional network with 50 layers that employs skip connections to mitigate the vanishing gradient problem. We used the standard ResNet50 architecture pre-trained on ImageNet.
2. DenseNet121 is a densely connected convolutional network with 121 layers, where each layer receives feature maps from all preceding layers. This architecture promotes feature reuse and reduces the number of parameters. The standard DenseNet121 pre-trained on ImageNet was adopted in our experiments.
3. EfficientNet-B3 is a scalable convolutional architecture that uniformly scales network depth, width, and resolution using a compound coefficient. We utilized the EfficientNet-B3 variant, which offers a good trade-off between accuracy and computational efficiency.
4. Vision Transformer (ViT-Base) divides input images into fixed-size patches and processes them using a standard Transformer encoder. We employed the ViT-Base model with a patch size of 16 (ViT-B/16), pre-trained on ImageNet-21k and fine-tuned on ImageNet-1k.
5. Swin Transformer (Tiny) is a hierarchical vision transformer that uses shifted windows to compute self-attention efficiently. We used the Swin-Tiny variant, which provides a balance between model capacity and computational cost while maintaining strong performance on vision tasks.

The performance of all evaluated models is summarized in **Table 4**. The proposed ConvNeXt V2 + CBAM model achieved the highest overall accuracy (95.00%) and F1-score (94.99%) among all architectures, outperforming both conventional convolutional networks (ResNet50, DenseNet121, and EfficientNet-B3) and transformer-based models (ViT-Base and Swin-Tiny). In terms of model complexity, the proposed model contains approximately 28.6 million parameters, which is comparable to Swin Transformer-Tiny and significantly lighter than ViT-Base (86 million parameters), while delivering superior accuracy. Although EfficientNet-B3 is more parameter-efficient (12 million parameters), it achieves lower performance than the proposed approach. This indicates that the proposed model offers a favorable trade-off between accuracy and model size. The superior performance of ConvNeXt V2 + CBAM can be attributed to several key factors. First, ConvNeXt V2 incorporates modern design elements, including large kernel convolutions and an inverted bottleneck structure, which enable more effective extraction of both fine-grained local textures and broader contextual information compared to traditional architectures such as ResNet and DenseNet. Second, the integration of the Convolutional Block Attention Module (CBAM) allows the model to explicitly model channel-wise and spatial interdependencies. This attention mechanism enables the network to dynamically emphasize diagnostically critical regions (such as polyp boundaries or areas of mucosal inflammation) while suppressing irrelevant background information — a capability particularly valuable in colonoscopic images where pathological features often occupy only a small portion of the frame. In contrast, while transformer-based models like ViT and Swin Transformer are powerful, they generally require larger amounts of training data to fully leverage their capacity. In this focused three-class medical imaging task, the attention-augmented ConvNeXt V2 architecture demonstrates better data efficiency and feature discriminability. Overall, the combination of ConvNeXt V2’s strong representational power with CBAM’s targeted attention results in more robust and clinically meaningful feature learning, leading to consistent improvements across all evaluation metrics. In addition to classification performance, the computational efficiency of the models was evaluated. As shown in **Table 4**, the proposed ConvNeXt V2 + CBAM model achieves the highest accuracy while maintaining competitive computational cost. Although it has a higher number of parameters than EfficientNet-B3, its FLOPs and inference time remain comparable to other CNN-based models and significantly lower than ViT-Base. These results indicate that the proposed model offers a favorable trade-off between accuracy and computational efficiency.

**Table 4:** Performance comparison of the proposed ConvNeXt V2 + CBAM model with other baseline architectures.

Model	Accuracy (%)	Precision (%)	Recall (%)	F1-score (%)	Parameters (M)	FLOPs (G)	Inference time (ms)
ResNet50	91.47	91.65	91.32	91.48	25.6	4.12	13.8
DenseNet121	92.35	92.48	92.21	92.34	8.0	2.88	11.5
EfficientNet-B3	93.82	93.95	93.71	93.83	12.0	1.84	9.7
ViT-Base (Patch 16)	92.68	92.81	92.54	92.67	86.0	17.58	24.6
Swin Transformer (Tiny)	93.95	94.12	93.81	93.96	28.3	4.52	14.2
ConvNeXt V2 + CBAM (Proposed)	95.00	95.07	94.92	94.99	28.6	4.68	12.4

To further validate the reliability and consistency of the proposed model, a statistical analysis was performed on the results obtained from 5-fold stratified cross-validation. As presented in **Table 5**, the proposed ConvNeXt V2 + CBAM model achieved a mean accuracy of 95.00 ± 0.82%, with a 95% confidence interval of [94.28%, 95.72%]. Similar consistency was observed for precision (95.07 ± 0.81%, 95% CI: [94.36%, 95.77%]) and F1-score (94.99 ± 0.82%, 95% CI: [94.27%, 95.71%]). The relatively low standard deviations across folds indicate stable model performance under different data partitions. To formally assess the significance of the performance improvements, paired Wilcoxon signed-rank tests were conducted using the per-fold metrics. The proposed model demonstrated statistically significant improvements over all baseline architectures (ResNet50, DenseNet121, EfficientNet-B3, ViT-Base, and Swin-Tiny) with p-values below 0.05. These results confirm that the observed performance gains are statistically meaningful and not attributable to random variation.

**Table 5:** Statistical summary of the proposed ConvNeXt V2 + CBAM model performance across 5-fold stratified cross-validation.

Metric	Mean ± Std	95% confidence interval	Standard error of mean
Accuracy	95.00 ± 0.82	[94.28, 95.72]	0.37
Precision	95.07 ± 0.81	[94.36, 95.77]	0.36
F1-Score	94.99 ± 0.82	[94.27, 95.71]	0.37

Values are reported as Mean ± Standard Deviation across five folds. All performance improvements over baseline models were statistically significant (p < 0.05, Wilcoxon signed-rank test).

## 5 Discussion

Here we compare the performance improvements of the proposed model with the baseline ConvNeXt V2 architecture and analyze the attention mechanism in detail. The visual similarity within disease classifications such as polyps and ulcerative colitis is significant. Colonoscopic images tend to have complex textures and subtle patterns. Such subtle distinctions may be missed by typical convolutional architectures. The proposed model can strengthen useful features and suppress irrelevant background information by integrating the CBAM attention module into the ConvNeXt V2 backbone. This allows for improved class identification which is particularly helpful in difficult situations where only small visual differences exist. To increase the generalizability of the model and capture robust characteristics that are not dependent on the data distribution, we exploit data augmentation and stratified cross-validation.

Spatial attention and channel attention help the network to learn the most important feature channels and the most relevant regions of a picture, respectively. This is particularly useful for the analysis of colonoscopic images, where clinically relevant areas such as polyps or inflammatory tissues often only occupy a tiny fraction of the picture. Feature extraction without attention mechanisms might pollute these regions with irrelevant information. The inclusion of CBAM in the network improves both the classification accuracy and prediction reliability by better focusing on these areas important for diagnosis. This adds more evidence to this, as all the folds have the same results.

**Figure 10:**
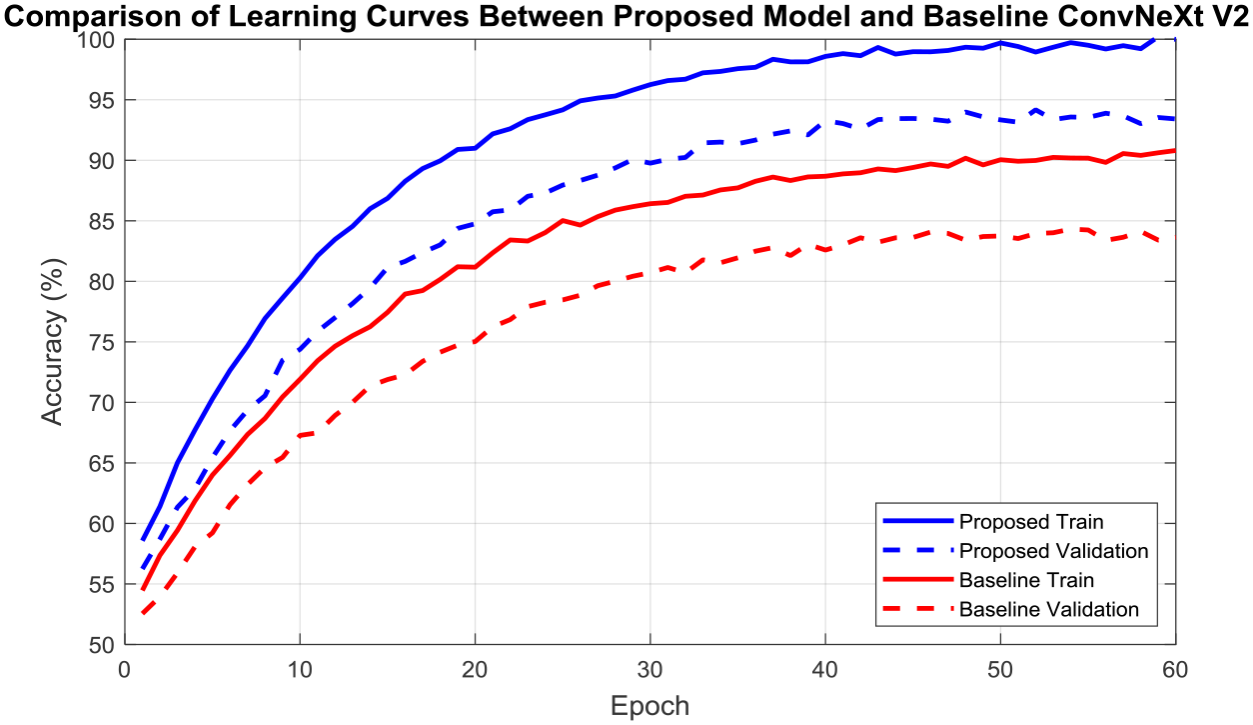
Comparison of training and validation accuracy between the baseline ConvNeXt V2 and the proposed model.

This indicates that the proposed model performs better not only in terms of accuracy but also in terms of precision and F1-score. This means that the model is useful to reduce both overall error and class specific error in misclassification. The results show that the integration of attention processes into modern convolutional architectures is especially well suited for medical image classification problems that require the differentiation of fine-grained features. The comparison of training and validation accuracy of baseline ConvNeXt V2 model and the proposed attention enhanced model is shown in **Figure 10**. As can be seen, the proposed model converges faster in the early training stages, implying more efficient feature learning. In addition, the training and validation curves of the proposed model do not have a huge gap, which makes less overfitting and better generalization. In contrast, the baseline model does slightly worse on validation and converges slower.

**Figure 11:**
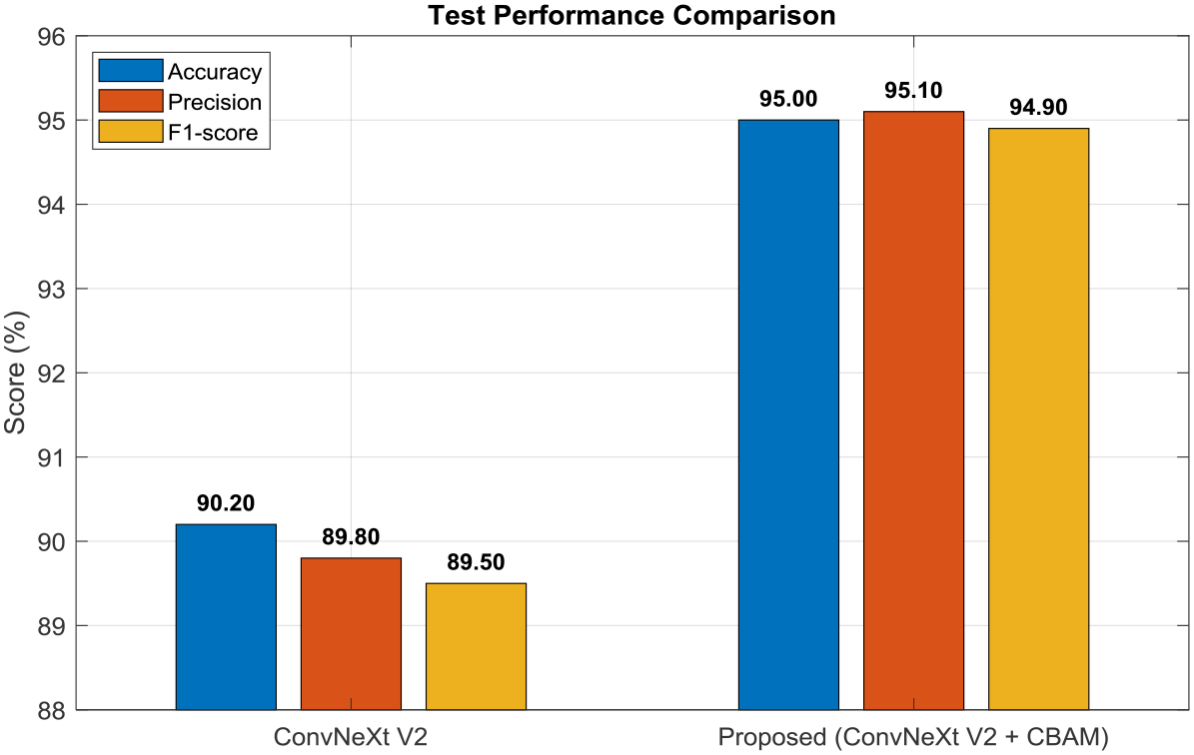
Performance comparison between the baseline ConvNeXt V2 and the proposed model in terms of accuracy, precision, and F1-score.

**Figure 11** shows comparison of the baseline and suggested models in terms of performance indicators such as accuracy, precision and F1 score. Clearly, the proposed model is superior to the baseline on every metric used for evaluation. In jobs like medical image classification, a 5% increase in accuracy is very meaningful. Improved trade-off between false positives and false negatives is also indicated by the accuracy and F1-score increment, which shows that the suggested model is better. The results show that the attention improved architecture is more accurate in classification and more efficient in feature extraction.

The external validation on HyperKvasir provides valuable evidence regarding the robustness of the proposed framework. Although a performance drop of 4.1–5.2% was observed relative to the internal Kvasir results, the model still achieved over 88% accuracy across all three classes on completely unseen data. This outcome indicates that the integration of the Convolutional Block Attention Module (CBAM) enables the model to focus on clinically relevant features even when faced with variations in imaging conditions and data distribution. The relatively smaller drop in cecum classification (3.8%) suggests that the model effectively learned stable representations of normal anatomical structures. In contrast, the larger drop in polyp detection highlights the inherent challenge of generalizing to diverse polyp appearances across different datasets. These findings support the potential applicability of the proposed attention-enhanced ConvNeXt V2 architecture in real-world clinical settings, while also underscoring the importance of external validation for assessing true generalization performance.

**Figure 12:**
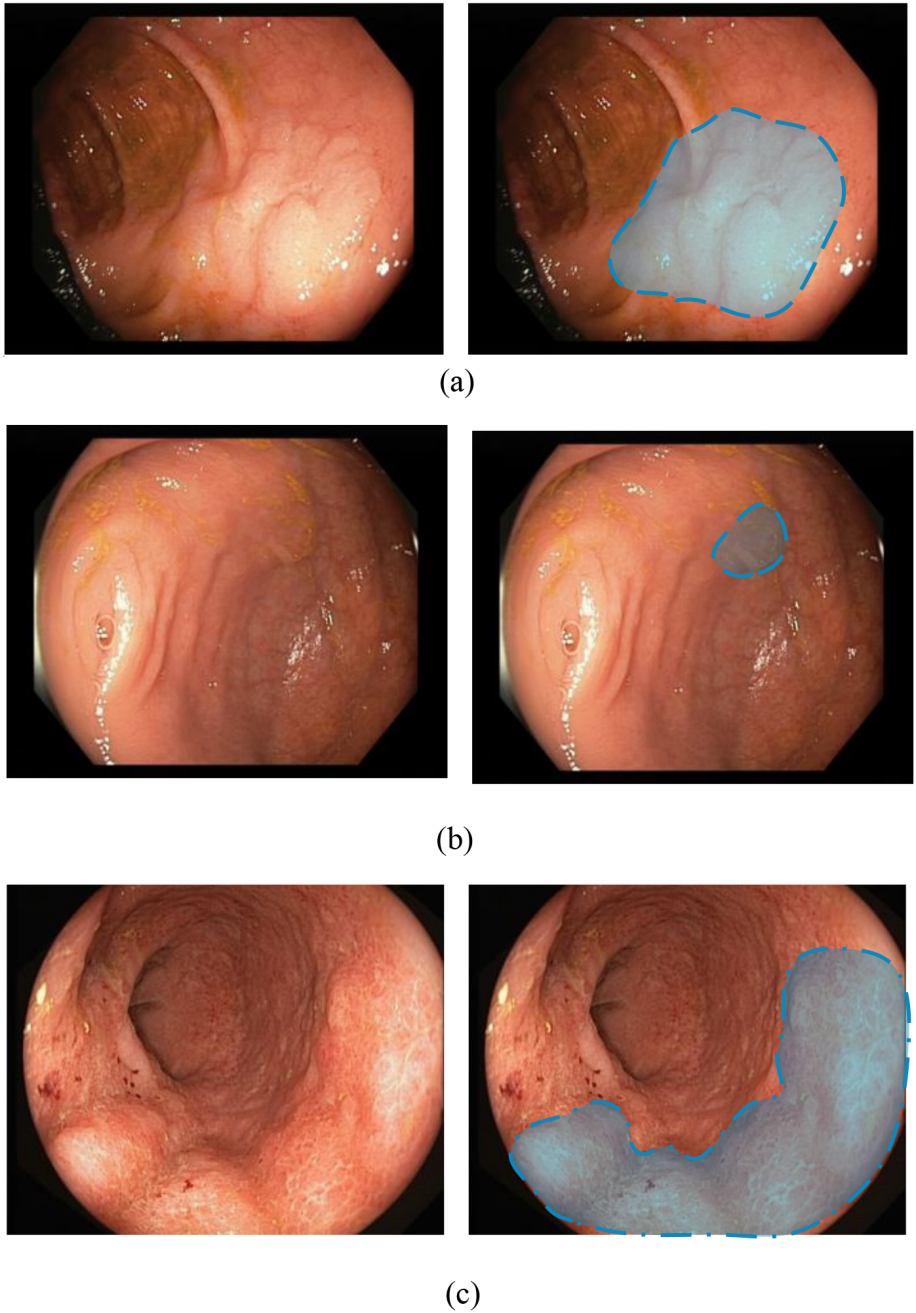
Qualitative error analysis of misclassified cases, **(a)** A polyp misclassified as ulcerative colitis, **(b)** Normal mucosa misclassified as a polyp, **(c)** Ulcerative colitis misclassified as a polyp.

To further investigate the model’s decision-making process and identify potential sources of error, a qualitative error analysis was performed on misclassified cases from the test set. As illustrated in **Figure 12**, the majority of misclassifications occurred between the polyp and ulcerative colitis classes, as well as between normal mucosa and polyp. In **Figure 12(a)**, a polyp was misclassified as ulcerative colitis. The lower portion of the lesion exhibited marked mucosal irregularity, erythema, and surface nodularity, which closely resembled the inflammatory changes typically observed in ulcerative colitis. These visual similarities likely contributed to the model’s confusion between the two pathological entities. In **Figure 12(b)**, normal colonic mucosa was incorrectly classified as a polyp. A prominent natural mucosal fold or lesion located in the upper region of the image was misinterpreted by the model as a polypoid structure. This highlights the challenge of distinguishing benign anatomical variations from true pathological lesions in colonoscopic images. In **Figure 12(c)**, ulcerative colitis was misclassified as a polyp. The presence of severe mucosal swelling, nodularity, and inflammatory exudate created a polyp-like morphology, leading the model to favor the polyp class over ulcerative colitis. These findings underscore the inherent visual overlap between different colorectal conditions and emphasize the need for more robust feature discrimination, particularly in cases with overlapping inflammatory and polypoid features.

Future work will explore the integration of multi-modal data to further enhance diagnostic performance. One potential direction involves combining image features extracted by the proposed model with textual embeddings derived from clinical reports and patient history using pre-trained language models. Early fusion or cross-attention mechanisms can be employed to enable effective interaction between visual and textual modalities, allowing the model to utilize complementary information that may improve diagnostic accuracy and clinical relevance. Additionally, future studies will explore the application of other explainable AI techniques, such as Grad-CAM++ and SHAP, to obtain more comprehensive and quantitative insights into the model’s decision-making process. Furthermore, to facilitate real-time deployment in clinical environments, future work will explore model optimization techniques such as quantization, pruning, and knowledge distillation. Quantization can reduce the model’s memory footprint and computational requirements by lowering the precision of weights and activations. Pruning can eliminate redundant parameters to create a more compact network, while knowledge distillation can transfer the learned knowledge from the current model to a smaller, more efficient student model. These approaches are expected to significantly improve inference speed while maintaining acceptable diagnostic accuracy, making the system more suitable for real-time clinical applications.

### 5.1 Limitations

This study has several limitations that should be acknowledged. First, although the proposed framework was evaluated on a clinically relevant three-class subset of the Kvasir dataset, the original dataset contains eight classes. The focused selection of cecum, polyp, and ulcerative colitis, while enabling balanced and rigorous evaluation, may limit direct applicability to broader multi-class gastrointestinal disease classification scenarios. Second, external validation was performed on only one independent dataset (HyperKvasir). Although the model demonstrated reasonable generalization with a moderate performance drop, further validation on multi-center and multi-vendor colonoscopy datasets is necessary to confirm robustness across diverse clinical environments. Third, interpretability analysis was primarily based on attention maps generated by the CBAM module. While these visualizations provide useful qualitative insights, the incorporation of additional explainable AI techniques such as Grad-CAM++ or SHAP could offer more comprehensive and quantitative understanding of the model’s decision-making process. Finally, the current study was conducted on static images with a fixed high resolution. Future work should explore the model’s performance on video sequences and investigate optimization strategies for real-time clinical deployment with lower computational overhead.

## 6 Conclusion

In this study, we proposed an attention-enhanced ConvNeXt V2 framework for the multi-class classification of colonoscopic images into three clinically relevant categories: normal (cecum), polyp, and ulcerative colitis. By integrating a Convolutional Block Attention Module (CBAM) into the ConvNeXt V2 backbone, the model achieved improved feature representation by focusing on diagnostically important regions. Using a balanced subset of the Kvasir dataset and 5-fold stratified cross-validation, the proposed model attained a mean accuracy of 95.00%, precision of 95.07%, and F1-score of 94.99%, significantly outperforming the baseline ConvNeXt V2 model. The incorporation of the attention mechanism enabled the model to effectively distinguish between visually similar classes by emphasizing subtle pathological features. These results demonstrate the potential of combining modern convolutional architectures with attention mechanisms for accurate and interpretable analysis of colonoscopic images. Future work will focus on extending the framework to larger and more diverse datasets, incorporating additional explainable AI techniques, and optimizing the model for real-time clinical deployment.

## Data Availability

Publicly available datasets were analyzed in this study. This data can be found here: https://www.kaggle.com/datasets/meetnagadia/kvasir-dataset.
